# Population variation in early development can determine ecological resilience in response to environmental change

**DOI:** 10.1111/nph.16453

**Published:** 2020-02-29

**Authors:** Greg M. Walter, Stefania Catara, Jon R. Bridle, Antonia Cristaudo

**Affiliations:** ^1^ School of Biological Sciences University of Bristol Bristol BS8 1TQ UK; ^2^ Department of Biological, Geological and Environmental Sciences University of Catania Catania 95128 Italy

**Keywords:** climate change, ecological resilience, environmental sensitivity, genotype‐by‐environment interactions, germination success, intraspecific variation, Mediterranean ecosystems, seed ecology

## Abstract

- As climate change transforms seasonal patterns of temperature and precipitation, germination success at marginal temperatures will become critical for the long‐term persistence of many plant species and communities. If populations vary in their environmental sensitivity to marginal temperatures across a species’ geographical range, populations that respond better to future environmental extremes are likely to be critical for maintaining ecological resilience of the species.
- Using seeds from two to six populations for each of nine species of Mediterranean plants, we characterized patterns of among‐population variation in environmental sensitivity by quantifying genotype‐by‐environment interactions (G × E) for germination success at temperature extremes, and under two light regimes representing conditions below and above the soil surface.
- For eight of nine species tested at hot and cold marginal temperatures, we observed substantial among‐population variation in environmental sensitivity for germination success, and this often depended on the light treatment. Importantly, different populations often performed best at different environmental extremes.
- Our results demonstrate that ongoing changes in temperature regime will affect the phenology, fitness, and demography of different populations within the same species differently. We show that quantifying patterns of G × E for multiple populations, and understanding how such patterns arise, can test mechanisms that promote ecological resilience.

## Introduction

Investigating how and when populations can persist in the face of ongoing rapid climate change is critical for understanding the resilience of ecological communities (Parmesan, 2006; Shaw & Etterson, 2012). For any given species, ecological resilience is likely to increase if different populations vary in their responses to environmental variation, and some populations are more likely to persist than others. Such persistence will likely require rapid changes in key life history traits, as shown by shifts to earlier spring growth in deciduous trees (Chmielewski & Rotzer, 2001) and earlier breeding date in birds (Charmantier *et al.*, 2008). Species can then persist through local adaptation or through expansion (migration or human‐assisted translocation) from resilient populations to more vulnerable populations (Angert *et al.*, 2011; Hoffmann & Sgrò, 2011). Quantifying patterns of population variation in environmental sensitivity for multiple species and key ecological traits can help to identify whether variation among populations of a species (intraspecific variation) can increase the ecological resilience the species in response to climate change (Kimball *et al.*, 2010; Cochrane *et al.*, 2015; Barga *et al.*, 2017).

Testing for among‐population variation in environmental sensitivity requires sampling individuals from different populations across a species’ distribution and quantifying their performance in response to environmental variation, especially to changes beyond existing environmental conditions. We expect to see overall differences among populations (G), and we expect that populations will show reductions in absolute performance when exposed to environmental variation that represents novel conditions in the future (E). However, if populations vary in the extent to which they show reduced performance in novel environments (i.e. some populations show a higher performance relative to others) we will observe an interaction between population and environment, known as G × E or genotype‐by‐environment interactions (Box 1; Kawecki & Ebert, 2004; Anderson *et al.*, 2011; Josephs, 2018). In addition, if populations perform relatively well at one temperature extreme but relatively poorly at the opposite temperature extreme, then different populations will have a higher relative performance and be more likely to persist, depending on how the environment changes. Quantifying G × E in laboratory experiments, therefore, allows us to to directly test for among‐population variation in the response to extreme temperatures and identify populations with high relative performance (and therefore higher resilience) in response to climate change.

Box 1 **Conceptual framework of genotype‐by‐environment interaction (G × E) applied to ecology and conservation biology**

For a given group of genotypes adapted to different environments, genotypes will perform better in their own environment when compared with alternative environments (Anderson *et al.*, 2011). This can be quantified as an interaction between genotype and the environment (i.e. a genotype‐by‐environment interaction, G × E), where genotype performance is dependent upon the environment in which they are tested (Ingleby *et al.*, 2010; Anderson *et al.*, 2011). This G × E conceptual framework has been used successfully to study whether populations for a given species are adapted to their local environment. By testing a range of populations in reciprocal transplant experiments and quantifying G × E for population performance, local adaptation exists where populations tested in their local environment perform relatively better than foreign populations (Kawecki & Ebert, 2004; Hereford, 2009). The G × E framework has also been used extensively in plant/animal breeding to identify genotypes for genetic improvement.

**Figure 1:**
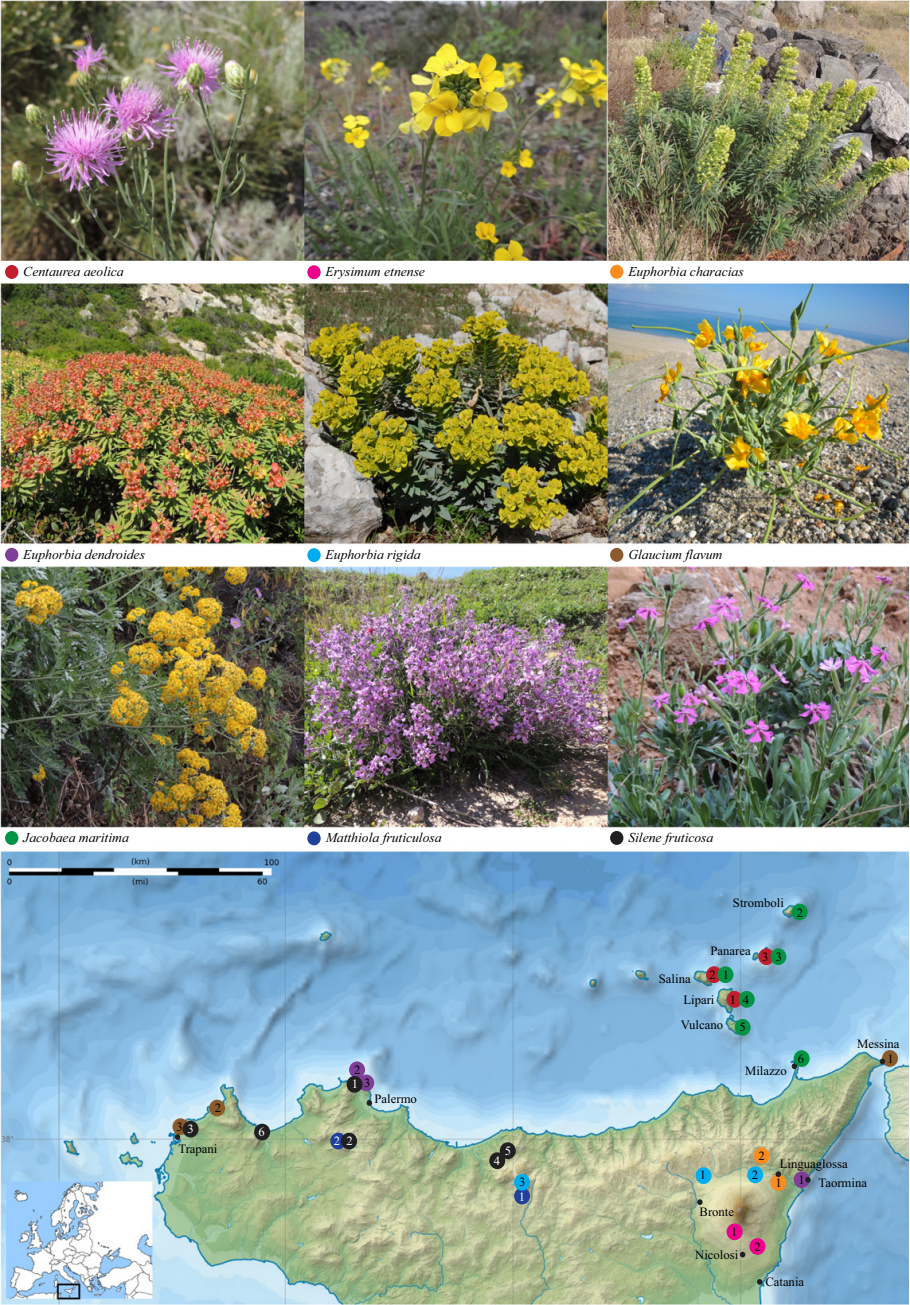
Photographs of the nine study species and the populations sampled across northern Sicily. Numbers match population numbers in Table 1. Inset shows the location in continental Europe. Modified from base map © Sémhur/Wikimedia Commons/CC‐BY‐SA‐3.0.

The G × E approach can be extended to answer important questions in ecology and conservation biology by testing the response of populations to conditions expected under climate change scenarios. G × E emerges when populations vary in their response to environmental variation as a change in the magnitude of among‐population variance, or a change in rank performance, between environments. Both types of G × E provide important information on species’ resilience (Ingleby *et al.*, 2010; El‐Soda *et al.*, 2014). Changes in variance at environmental extremes are expected if genotypes experience novel environmental conditions and either respond erratically, increasing variance (e.g. response to hot temperatures in Box Fig. 1a,b; Badyaev, 2005; Ghalambor *et al.*, 2007), or respond similarly to a stressful environment, reducing variance (e.g. response to cold temperatures in Box Fig. 1a,b; Hoffmann & Merila, 1999; Charmantier & Garant, 2005; Charmantier *et al.*, 2008). By contrast, G × E observed as a change in relative performance between environments is indicative of performance trade‐offs between different environmental extremes (Box Fig. 1c,d). In this scenario, environmental change should expose trade‐offs in performance (Hereford, 2009; Agrawal *et al.*, 2010), suggesting that populations will respond differently depending on the direction of environmental change.

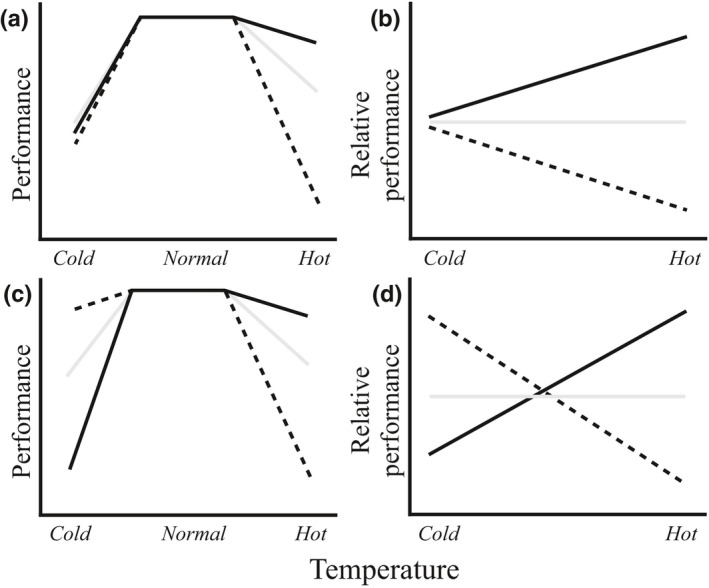

**Box Fig. 1** Schematic diagram describing genotype‐by‐environment (G × E) interactions in response to marginal environments. In this example, we assess the response of three populations, represented by the three different lines, to an environmental gradient (here, temperature). (a, c) Changes in absolute performance with temperature for two scenarios of G × E. Populations perform similarly within the typical ‘normal’ temperature range, but there is reduced performance with marginal temperatures (‘cold’ and ‘hot’), where differences among the populations also emerge. (b, d) The same patterns of G × E, but only for the extreme temperatures, and relative to the mean performance in each temperature extreme. This quantifies the interaction between population performance and temperature to estimate the difference in relative performance of the populations at each environmental extreme. G × E interactions can occur as increases/decreases in the among‐population variance between the temperature extremes (a, b) or as changes in relative performance (c, d), where populations change rank between the temperature extremes.

Quantifying G × E for multiple populations of a species provides a framework that can identify the populations that perform well at environmental extremes (e.g. hot temperatures) relative to the other populations, even if the overall trend is a reduction in performance (Box Fig. 1; see also Stratton, 1994). This experimental framework can also identify whether high relative performance at one extreme is associated with lowered performance at the opposite extreme, creating a performance trade‐off between temperature extremes (e.g. hot vs cold temperatures in Box Fig. 1d; Agrawal *et al.*, 2010). In addition, combining patterns of G × E quantified in the laboratory with data for environmental variables from the sampling locations of the populations can provide information on how among‐population variation in environmental sensitivity arises. If, for example, populations from warmer locations of a species range perform relatively better at warmer temperatures in the laboratory than those from colder locations, then natural environmental variation has likely created variation among populations in environmental sensitivity.

Germination responses to marginal environments are a particularly important factor affecting seedling recruitment and population persistence in response to climate change (Donohue *et al.*, 2010; Cochrane, 2016; Jiménez‐Alfaro *et al.*, 2016). For species that occupy highly predictable seasonal environments, moisture availability during unseasonal temperature extremes can prevent germination (Hills & van Staden, 2003; Narbona *et al.*, 2007). This will reduce future viability, with important consequences for recently generated seeds as well as those found below the soil surface (Ooi *et al.*, 2009; Walck *et al.*, 2011). However, if climate change results in rainfall events that coincide with extreme hot or cold temperatures, and multiple populations of a given species vary in their germination tolerance of such extremes, then populations that are able to germinate in suboptimal temperatures will help increase ecological resilience of the species by persisting locally. We still do not understand whether specific patterns of intraspecific variation in germination performance can increase the ecological resilience of species to climate change (Cochrane *et al.*, 2015), or whether such patterns are driven by variation in the natural environments (Cochrane *et al.*, 2014; Barga *et al.*, 2017; Chamorro *et al.*, 2017; Fang *et al.*, 2017; Yeşilyurt *et al.*, 2017). In a laboratory setting, testing the germination performance of seeds from different populations across a range of temperatures that represent conditions at, and outside, their ecological margins can quantify intraspecific variation in environmental sensitivity as population‐by‐temperature interactions (analogous to G × E).

The Mediterranean region is a biodiversity hotspot with exceptional levels of endemism and is likely to be particularly sensitive to climate change (Giorgi & Lionello, 2008; Lionello & Scarascia, 2018). Mediterranean plants are often restricted to small habitat refuges along the coastline, small islands, or to narrow elevational ranges on mountains. Cold, wet winters and hot, dry summers characterize the Mediterranean climate, providing two periods when temperature and water availability are suitable for germination (spring and autumn) (Lloret *et al.*, 2004; Cochrane *et al.*, 2011). Given current forecasts of climate change are for more variable temperatures and reduced precipitation across the Mediterranean region, suboptimal temperatures are likely to impinge on these ideal germination periods (Walck *et al.*, 2011; Hadjou Belaid *et al.*, 2018). More specifically, if seasonal rain arrives in summer or winter, or at a time when the new climate regime makes extreme temperatures more common, plants in these habitats will need to germinate and grow under marginal temperatures if they are to persist. Mediterranean ecosystems are therefore important model systems to test whether local sensitivities to the environment can confer resilience across the range of a species (Lavergne *et al.*, 2004; Thompson *et al.*, 2005).

For nine species of Mediterranean plants, we quantified the variation among populations in their germination sensitivity to marginal temperatures, which we then related to climatic patterns in the natural environments. We collected seeds from individuals at two to six locations for each species, which are endemic or native to the Mediterranean Basin. The nine species are from seven genera (*Centaurea*, *Erysimum*, *Euphorbia*, *Glaucium*, *Jacobaea*, *Matthiola*, and *Silene*), and five plant families (Asteraceae, Brassicaceae, Euphorbiaceae, Papaveraceae and Caryophyllaceae). Generally, these species are woody or herbaceous perennials that occupy patches of habitat that are geographically small and restricted, which, combined with their reduced dispersal and limited dormancy, mean it will be difficult for populations to expand or shift. Under laboratory conditions, we exposed seeds of all species to a range of germination temperatures that represent conditions within and outside their normal ecological conditions. For each temperature, we also exposed seeds to photoperiod treatments that represented the soil surface (dark only) and above the soil surface (dark/light). To estimate population variation in environmental sensitivity we quantified patterns of G × E in germination success for temperature extremes. We predicted that, if variation in environmental sensitivity could promote the persistence of certain populations, different populations would perform better at different extremes (hot vs cold) and we would observe substantial G × E for germination success. We then compared patterns of G × E with climate data to test whether climate determined intraspecific variation for environmental sensitivity.

## Materials and Methods

### Study species and seed collection

We studied nine species of Mediterranean plants: *Centaurea aeolica* Lojac., *Erysimum etnense* Jordan, *Euphorbia characias* L., *Euphorbia dendroides* L., *Euphorbia rigida* M. Bieb., *Glaucium flavum* Crantz, *Jacobaea maritima* (L.) Pelser & Meijden subsp. *bicolor* (Willd.) B. Nord. & Greuter, *Matthiola fruticulosa* (L.) Maire subsp. *fruticulosa*, and *Silene fruticosa* L. Detailed descriptions of the habitat and ecology for all species are provided in Table 1, and detailed locations of populations are given in Supporting Information Table S1. In terms of dormancy, under natural conditions, the three *Euphorbia* species have low physiological dormancy (nondeep dormancy), meaning they are dormant only for a short period after ripening (Cristaudo *et al.*, 2019), probably to minimize germination during the hot summer months (del Cacho *et al.*, 2012). Dormancy in the remaining species is also low or absent, because seed dispersal occurs during the dry summer, preventing germination until water is present. Low dormancy means that seeds of our study species do not form a persistent seed bank and germination is controlled by the environment rather than by seed dormancy. Seed dispersal ability is also low (Table 1), with wind dispersal in only one species (*J. maritima*). When stored in the laboratory, seeds of these species maintain their viability for several years.

**Table 1:** Taxa and populations used in the study.

Taxa (family)	Habitat	Biology and life history	Seed dispersal	Population	Harvest date	Exp. date
*Centaurea aeolica* (Asteraceae)	Cliffs and dry slopes of volcanic origin	Chamaephyte. Regrow from rootstock each autumn	Fleshy seeds dispersed by gravity or myrmecochory	1. Lipari	13 Jun; 14 Jul	Mar 15
				2. Salina	14 Jul	
				3. Panarea	13 Jun	
*Erysimum etnense* (Brassicaceae)	Dry and stony areas, old lava flows, road edges	Regrow from rootstock each autumn	Small lightweight seeds likely dispersed by gravity	1. Mt Vetore	15 Sep	16 Jan
				2. Ragala	15 Sep	
*Euphorbia characias* (Euphorbiaceae)	*Quercus ilex* woodland, scrubland, garigue	Deciduous nanophanerophyte. Leaves die in summer, regrow in autumn	Large hardened seeds dispersed by explosive capsules and myrmecochory	1. Linguaglossa	12 May	14 Feb
				2. Francavilla di Sicilia	12 May	
*Euphorbia dendroides*	Rocky limestone slopes, mostly near the sea			1. Castelmola	12 May	14 Feb
				2. Capo Gallo	13 May	
				3. Mt Pellegrino	13 May	
*Euphorbia rigida*	Stony slopes and dry riverbeds	Chamaephyte. Regrow from rootstock each autumn		1. Bronte	12 May	14 Feb
				2. Castiglione di Sicilia	12 May	
				3. Isnello	13 Jun	
*Glaucium flavum* (Papaveraceae)	Shingle beaches, coastal cliffs, and sand dunes	Short‐lived perennial	Small black seeds dispersed by gravity and rain	1. Messina	12 Jul	13 Jan
				2. Cofano	12 Jul	
				3. Erice	12 Jul	
*Jacobaea maritima* subsp*. bicolor* (Asteraceae)	Coastal cliffs and rocky sites	Long‐lived perennial	Small elongated seeds with a pappus that are wind dispersed	1. Salina	13 Aug	16 Sep
				2. Stromboli	15 Jul	
				3. Panarea	13 Jul	
				4. Lipari	13 Jul	
				5. Vulcano	15 Jul	
				6. Milazzo	13 Jul	
*Matthiola fruticulosa* (Brassicaceae)	Clayey or marly slopes	Chamaephyte. Regrow from rootstock each autumn	Small lightweight seeds likely dispersed by gravity	1. Quacella	13 Jul	14 Jul
				2. Cozzo di Fratantoni	13 Jul	
*Silene fruticosa* (Caryophyllaceae)	Limestone cliffs	Chamaephyte. Low‐growing herb among rocks	Small black seeds dispersed by gravity, and assisted by wind	1. Mt Pellegrino	12 Jun	15 Mar
				2. Cozzo di Fratantoni	12 Jun; 13 Jun	
				3. Erice	12 Jun	
				4. Collesano	13 Jul	
				5. Gratteri	13 Jul	
				6. Mt Inici	13 Jun	

Specific locations of populations are found in Supporting Information Table S1.

Exp., experiment.

For each species, we collected seeds from two to six populations across Sicily and the Aeolian Islands (Italy) between 2012 and 2015 (Fig. 1; Table 1). Seed collections were planned according to protected areas legislation. Seeds were collected from all plants at each population on the same date, with each population containing 50 or more plants distributed in an area no larger than 1000 m^2^. Before being used in the germination experiments outlined below, collected seeds from each population were randomized and stored in paper bags at ± 25°C and 40–50% relative humidity at the University of Catania. Data from the experiments outlined below are located in Dataset S1.

### Experimental set‐up

Laboratory experiments allow traits in multiple populations to be measured in environmental conditions that characterize conditions both within and outside ecological margins. Therefore, despite not replicating natural conditions perfectly, performing germination tests at constant temperatures allows us to compare the ‘thermal window’ (between the minimum and maximum temperatures) of germination for multiple populations of a given species. In order to quantify environmental sensitivities of multiple populations for nine species, we were restricted to using stored seeds. Although freshly collected seeds may vary slightly in their performance, this is unlikely to affect the results of the study because our primary goal was to test for differences among populations. By contrast, storing seed meant that seeds could be kept viable for a long period of time, allowing populations and species to be assessed simultaneously, providing much more powerful comparative tests than would have been possible if seeds were tested sequentially and immediately after collection. In addition, and except where specifically stated, seeds from all populations were collected, stored, and used in experiments at the same time, and in the same manner, which minimized any systematic effects of seed storage on our comparisons.

Using a total of 40 705 seeds, we conducted germination experiments in controlled‐temperature growth cabinets between 2013 and 2016. We tested all species at 3°C, and at 5°C intervals between 5°C and 35°C. These temperatures were chosen to include optimal germination temperatures for Mediterranean plants (*c.* 15°C) while including extreme hot and cold temperatures. For each temperature treatment, seeds were tested in two light treatments: continuous darkness (D), representing seeds found below the soil surface; and in an alternating light/dark (L/D) regime with a 12 h : 12 h photoperiod representing seeds found above the soil surface. Seeds were not scarified for any species, but the pappus was removed from seeds of *J. maritima*. Seeds were randomized and placed on 9 cm diameter plastic Petri dishes containing three sheets of filter paper (Munktell grade 6–80 g m^−2^), which was kept moist throughout the experiment.

For each species, four Petri dishes were used to replicate each population for each treatment combination, with 25 seeds placed on each Petri dish (*n* = 100 seeds for each population in each treatment combination). All Petri dishes were wrapped with Parafilm M^®^ to reduce water loss and then randomly allocated to different growth cabinets (model MLR‐351H; Sanyo, Tokyo, Japan) with cool‐white fluorescent tubes (Osram FL 40 SS W/37). Each cabinet was maintained at a specific temperature regime, and digital thermometers were used to ensure consistency at the assigned temperature. For continuous darkness treatments, Petri dishes were immediately wrapped in two layers of aluminium foil and placed alongside the Petri dishes exposed to the alternating light regime. Germination was checked daily for the duration of the experiment (30 d for all species), and seeds were counted as germinated when they produced a root > 2 mm. Seeds in the dark photoperiod were checked at the end of the experiment to avoid light contamination. Petri dishes were rotated daily to ensure they were randomized with respect to microenvironmental variation. Germination success was recorded as a binary trait (0 = failed to germinate; 1 = seed germinated).

### Statistical analysis

We analysed germination success as a binary trait by implementing generalized linear mixed models with a logit link function, which quantified the probability of germinating in different temperature and light treatments. To compare germination performance across multiple temperature treatments we used a character state approach, which used temperature as a discrete variable. We chose this approach because our primary goal was to quantify variation among populations at marginal temperatures. By treating temperature as a discrete variable we could estimate the among‐population variance at specific marginal temperatures as well as the change in variance between hot and cold extremes, which provided the best test for G × E between temperature extremes.

**Figure 2:**
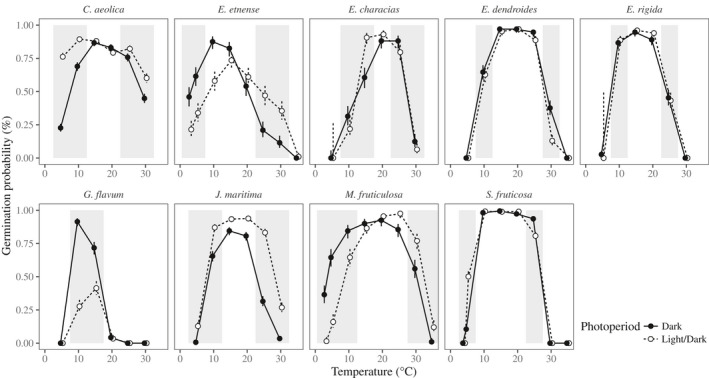
Germination probability for nine species (*Centaurea aeolica*, *Erysimum etnense*, *Euphorbia characias*, *Euphorbia dendroides*, *Euphorbia rigida*, *Glaucium flavum*, *Jacobaea maritima*, *Matthiola fruticulosa*, and *Silene fruticosa*) under different temperatures for continuous dark (closed circles with solid lines) and 12 h : 12 h, light : dark photoperiod (open circles with broken lines). Error bars represent 95% confidence intervals, and grey shading represents the marginal temperatures tested in subsequent analyses. *Glaucium flavum* showed very strict temperature‐dependent germination, meaning we focused on the central temperatures for this species.

To test for G × E in nine Mediterranean plant species, we conducted three comparisons. First, we compared germination performance under all temperatures, and for different light treatments. For each species, we tested whether germination success across temperature depended on whether seeds were located below (D treatment) vs above the soil surface (L/D treatment). Using the performance across all temperatures, we then identified the marginal temperatures as the most extreme cold and hot temperatures that showed reduced germination (generally, > 10% reduction compared with temperatures that showed maximum germination), but that were also nonzero (see grey shading in Fig. 2). We focused on the marginal temperatures because they represent the conditions at the edge of, or outside, normal germination conditions. Preliminary analyses showed that the choice of temperatures did not affect our results, because populations performed similarly well at all temperatures within each temperature extreme (i.e. they rarely showed changes in relative performance within a temperature extreme). Second, we used the marginal temperatures to test for population variation in germination success by quantifying G × E between temperature extremes. Third, we compared patterns of G × E with local climate data extracted from the WorldClim database (Fick & Hijmans, 2017) to identify whether climate predicted patterns of G × E for each species. All analyses were implemented using R v.3.6.1 (R Core Team, 2019).

#### Effect of light and temperature on germination success

For each species, we first analyzed the combination of light and temperature using generalized linear mixed models implemented using the R package lme4 (Bates *et al.*, 2015).

(Eqn 1) $$y_{\mathrm{ijkl}}=T_{i}+L_{j}+T_{i}\times L_{j}+d_{k(ij)}+e_{l(ijk)}$$

where *T_i_* represents the *i*th temperature, *L_j_* the *j*th light treatment and $T_{i}\times L_{j}$ the interaction between light and temperature treatments. We included Petri dish $d_{k(ij)}$ as the only random effect, and $e_{l(ijk)}$ represented the model error. We implemented Eqn 1 for each species separately using germination success as a binary response variable $y_{\mathrm{ijkl}}$‍. We tested the significance of the interaction term for each implementation using the log‐likelihood ratio between models with or without the interaction term. From this analysis, we quantified whether light–temperature interactions affected germination for each species.

Analyses using Eqn 1 also identified the temperatures associated with marginal (suboptimal) germination conditions for each species, representing the environmental extremes for germination performance. These marginal temperatures were used in the following analyses to understand population variation in germination performance (see grey shading in Fig. 2). We ignored temperatures that showed zero germination across all replicates for a given species because they represent the physiological (and ecological) limit to germination, and in this situation there is no information on differences among populations.

#### Population variation in germination at temperature extremes

To test for among‐population variation in germination sensitivity, we quantified G × E for population performance for the marginal temperatures. To quantify among‐population variance, we would ideally treat population as a random effect and temperature as a fixed effect, so that population represents a random sample from the broader geographical distribution. We can then test for G × E as a significant negative correlation between marginal hot and cold temperatures (representing temperature extremes for germination success). However, random effects should only be used with sample sizes of at least five (Bolker *et al.*, 2009), which was only possible for two of our sampled species (population *n* = 6 for both *J. maritima* and *S. fruticosa*).

Estimation of covariance matrices for random effects for these two species was conducted using a Bayesian framework, where we could quantify the error by estimating the posterior distribution for each parameter. For *J. maritima* and *S. fruticose*, we implemented the generalized linear mixed model with the R package mcmcglmm (Hadfield, 2010).

(Eqn 2) $$y_{\mathrm{ijkl}}=T_{i}+p_{j}+d_{k(ij)}+e_{l(ijk)}$$

where $T_{i}$ represents the *i*th temperature as a fixed effect and $d_{k(ij)}$ the replicate Petri dish as a random effect. We included population $p_{j}$ as a random effect, for which we specified an unstructured covariance matrix to estimate population‐specific random intercepts for each temperature. $p_{j}$ then estimated an n×n covariance matrix (where *n* is the number of temperatures) representing the population variance within each temperature, and population covariance between each pairwise comparison of temperatures. A significant negative correlation between temperatures provides evidence for G × E, as trade‐offs for germination success, among populations. We implemented Eqn 2 independently for each species and separately for the two light treatments. Details of model implementation and convergence checks are located in Methods S1.

For the remaining species (where fewer than five populations were sampled), we quantified G × E by including populations within species as a fixed effect. To do so, we used lme4 to implement Eqn 1, replacing light treatment *L_j_* with population within species *P_j_* as a fixed effect. This model then estimated G × E as the interaction between temperature and population, within each species. We conducted separate analyses for the two light treatments. In several cases, we sampled populations within species in different years (Table 1). For these populations we included year sampled in the analyses, to test whether sampling year affected the size of significance of G × E.

#### Associating climate with population variation in germination performance

To test whether among‐population variation in climate described patterns of G × E for each species, we extracted climate data from WorldClim (Fick & Hijmans, 2017), which we compared with patterns of G × E from the previous section. We extracted the temperature of the wettest quarter (resolution of 2.5) for all 30 sampling locations in Table 1, representing the temperature during natural germination conditions. To quantify the pattern of G × E, we calculated the change in relative performance between temperature extremes by dividing each temperature by the mean performance and estimating the difference in relative performance between hot and cold temperatures. We then compared temperature in the natural habitats with the relative change in population performance between extreme hot and cold temperatures (G × E) using linear regression. We predicted that if climate described patterns of G × E, then populations from warmer locations would show higher relative performance at higher temperatures.

## Results

### Effect of light and temperature on germination success

All species (except *E. rigida*) showed high variation in germination sensitivity to temperature that depended on the light treatment (Fig. 2; Table S2). This often meant that one light treatment exhibited higher germination at both temperature extremes (e.g. light/dark photoperiod in *C. aeolica*; Fig. 2). However, three species (*E. etnense*, *M. fruticulosa* and *S. fruticosa*) showed performance trade‐offs, indicated as changes in relative performance (between hot and cold temperatures) for the L/D vs D photoperiod (Fig. 2).

### Population variation in germination at temperature extremes

**Figure 3:**
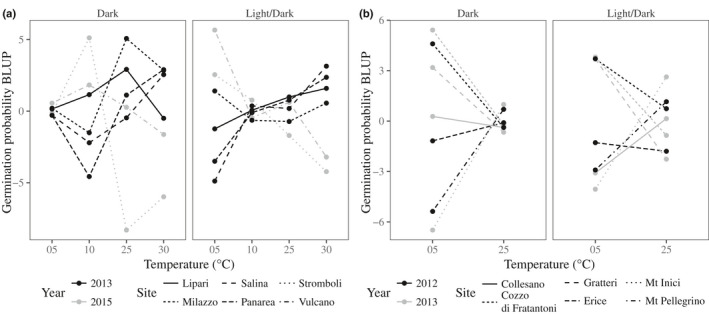
Estimates of germination performance at marginal temperatures for all populations sampled (dashed lines) for (a) *Jacobaea maritima* and (b) *Silene fruticosa*. Trade‐offs in germination performance are evident when populations differ in slope, suggesting that different populations perform better at opposite temperature extremes.

For *S. fruticosa* and *J. maritima*, we found significant negative correlations (ranging from −0.53 to −0.85) between hot and cold marginal temperatures, which were consistent for both photoperiod treatments (Table 2). Therefore, populations within both species exhibited trade‐offs in germination performance between hot and cold marginal temperatures, which are visualized as a change in relative performance between temperature extremes (Fig. 3). For *S. fruticosa*, among‐population variance in germination success was much greater in the cold than hot temperature in the dark‐only treatment, but not in the other light treatment. Differences between sampling years were not significant (grey vs black lines in Fig. 3). To confirm this, we reanalysed the data for each sampling year separately by implementing Eqn 2, estimating G × E as a fixed effect. This showed that the overall trends of trade‐offs remained (see Fig. S1), suggesting that trade‐offs between hot and cold temperature extremes were created by differences among populations and not due to an effect of sampling year.

**Table 2:** Quantifying genotype‐by‐environment interaction (G × E) using variance–covariance matrices showed significant negative correlations between extreme hot and cold temperatures for both (a) *Jacobaea maritima* and (b) *Silene fruticosa.*

(a)				
	5°C	10°C	25°C	30°C
Dark				
5°C	**2.4 (0, 7.44)**	0.06 (−0.97, 1)	0.15 (−0.93, 1)	0 (−0.99, 1)
10°C	1.29 (−8.89, 11.96)	**17.32 (1.82, 47.03)**	−0.51* (−0.96, 0.21)	−0.85* (−1, −**0.49)**
25°C	1.46 (−8.61, 12.57)	−11.62 (−42.69, 9.8)	**31.4 (1.74, 87.37)**	0.66* (−0.09, 0.99)
30°C	0.28 (−9.1, 8.85)	−12.93 (−38.96, 2.45)	17.08 (−4.46, 60.29)	**21.16 (0, 73.68)**
Light : dark				
5°C	**21.78 (1.34, 58.88)**	−0.26 (−1, 0.82)	−0.19 (−0.96, 0.69)	−0.82* (−1, −**0.49)**
10°C	−0.32 (−8.4, 8.68)	**1.13 (0, 3.92)**	0 (−0.89, 0.85)	0.03 (−0.93, 0.94)
25°C	−1.13 (−11.05, 8.75)	0.07 (−2.17, 2.19)	**2.42 (0.06, 6.97)**	0.38 (−0.61, 1)
30°C	−12.93 (−35.96, −1.09)	0.04 (−6.53, 4.76)	2.62 (−4.42, 10.86)	**13.21 (0.34, 34.12)**

(b)				
	5°C	25°C		
Dark				
5°C	**38.96 (1.68, 125.1)**	−0.68* (−1, 0.23)		
25°C	−5.14 (−19.67, 2.1)	**1.64 (0, 5.93)**		
Light : dark				
5°C	**16.02 (3.18, 43.21)**	−0.53* (−0.99, 0.09)		
25°C	−4.99 (−16.06, 4.32)	**5.11 (0.74, 12.83)**		

Numbers on the diagonal (in bold) represent population variance in germination success for each temperature. Off‐diagonal numbers represent G × E as population covariance among temperatures below the diagonal, and population correlations among temperatures above the diagonal (correcting for variance at each temperature). Numbers in parentheses represent the lower and upper 95% highest posterior density intervals. Correlations are significant in a Bayesian framework when greater than 90% of the posterior distribution (denoted by asterisks) does not overlap zero.

**Figure 4:**
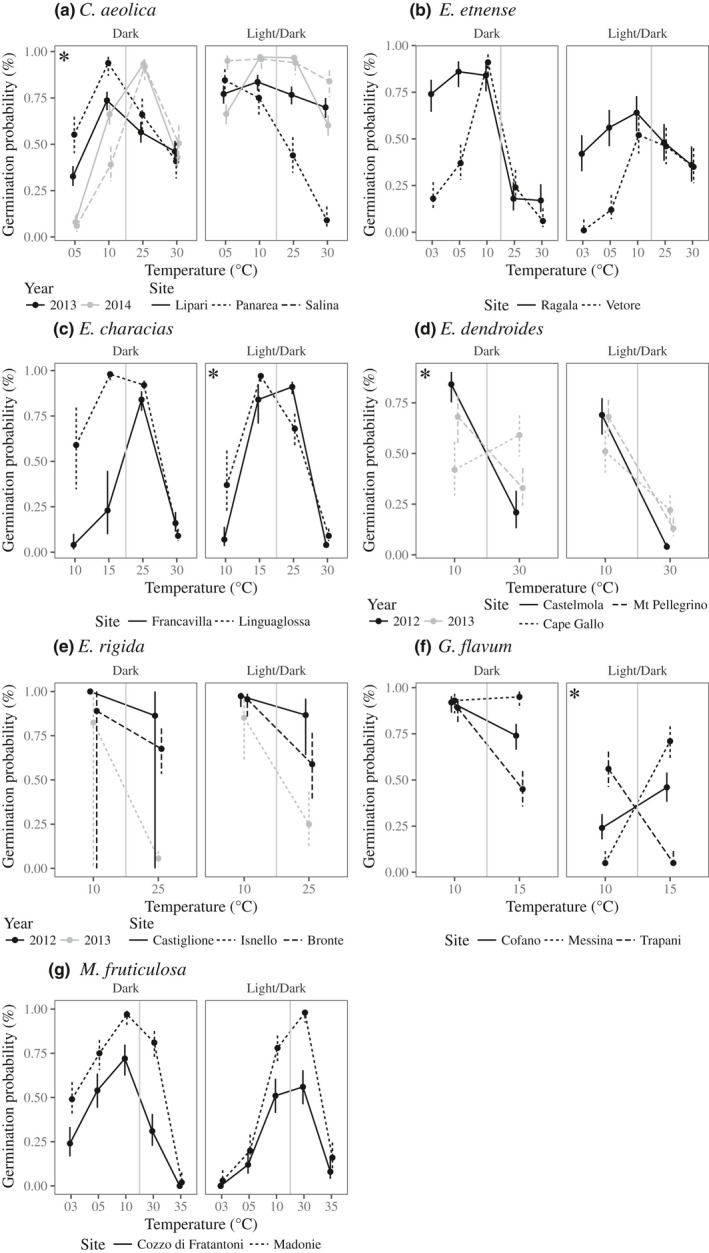
Genotype‐by‐environment interaction (G × E) for species sampled with fewer than five populations: (a) *Centaurea aeolica*; (b) *Erysimum etnense*; (c) *Euphorbia characias*; (d) *Euphorbia dendroides*; (e) *Euphorbia rigida*; (f) *Glaucium flavum*; (g) *Matthiola fruticulosa*. Vertical grey lines represent the split between the marginal cold and hot temperatures. Error bars represent 95% confidence intervals. Asterisks represent the tests where populations show trade‐offs between hot and cold temperatures.

For all species sampled with fewer than five populations, we found significant G × E on germination success (Fig. 4; Table S2). Changes in the magnitude of among‐population differences between temperature extremes were evident in *E. etnense* and *E. characias*, where differences among populations were greater at cooler temperatures, and also for *E. rigida* and *G. flavum*, which showed stronger differences at warmer temperatures (Fig. 4). Changes in relative germination performance (population rank) between hot and cold temperatures indicate a trade‐off where different populations perform significantly better at different extreme temperatures. We found evidence of such trade‐offs between hot and cold marginal temperatures for four species (*C. aeolica*, *E. characias*, *E. dendroides* and *G. flavum*). However, these trade‐offs were only present for one photoperiod treatment (Fig. 4). Of the three species that did not exhibit trade‐offs between marginal temperatures, two species (*E. etnense* and *M. fruticulosa*) exhibited light‐dependent germination sensitivity to temperature, as discussed earlier (Fig. 2). Furthermore, only *C. aeolica* exhibited variation between sampling years and not between populations within years (Fig. 4a).

### Associating climate with population variation in germination performance

**Figure 5:**
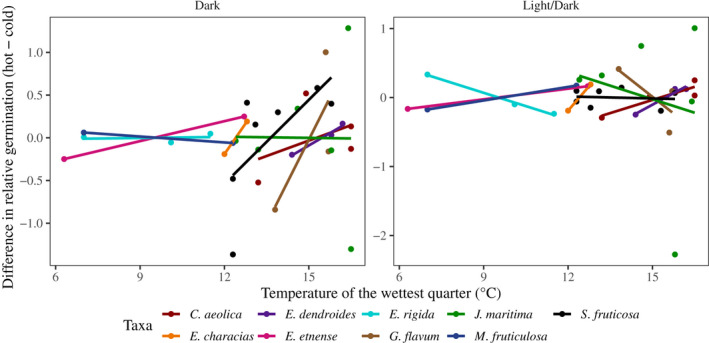
Associating natural environmental conditions with patterns of genotype‐by‐environment interaction (G × E) for all nine species (*Centaurea aeolica*, *Erysimum etnense*, *Euphorbia characias*, *Euphorbia dendroides*, *Euphorbia rigida*, *Glaucium flavum*, *Jacobaea maritima*, *Matthiola fruticulosa*, and *Silene fruticosa*). Overall, species showed tendencies for positive trends, suggesting sites from warmer areas performed relatively better at warmer temperature extremes but relatively worse at lower temperature extremes.

We tested whether among‐population differences in climate were associated with differences in G × E patterns (from the previous section). For the two species with six sites sampled, there were no strong relationships between climate of origin and G × E (Fig. 5). Only *S. fruticosa* exhibited a marginally significant positive relationship, and only in the dark photoperiod (ANOVA *F*_1,5_ = 4.083,* P *= 0.0993). This suggested that seeds from warmer sites performed relatively better at higher temperatures, for this comparison. The remaining species showed a general trend of positive relationships (Fig. 5), further suggesting that increased local temperature during ideal germination conditions also increased germination capacity for warmer laboratory conditions but reduced relative germination for cooler extreme temperatures.

## Discussion

Our results reveal strong and statistically significant patterns of G × E interactions within species for germination performance in the laboratory. Specifically, G × E was observed as changes in the magnitude of among‐population variance at temperature extremes and as performance trade‐offs between temperature extremes. Many of these patterns were driven by the L/D regime, suggesting that differences in seed behaviour depend on whether seeds are above or below the soil surface. The pattern and strength of G × E were dependent on the species, the photoperiod, and, for one species, the year seeds were collected (*C. aeolica*). Therefore, germination success among populations (within species) varied depending on the direction and magnitude of the changes in temperature and light. These results provide strong evidence that conservation approaches need to understand (and maintain) variation among populations in order to maximize the resilience of species and ecological communities to environmental change.

Photoperiod had strong species‐specific effects on germination success at temperature extremes. The two species *E. etnense* and *M. fruticulosa* germinated better in the dark at cold extreme temperatures, whereas *S. fruticosa* showed the opposite response, and several species showed a preference for one light treatment over the other. This suggests that responses to environmental change will likely depend on whether seedling recruitment relies on seeds germinating from below (D only) vs at the soil surface (L/D). Although the light treatments used are a simplification of natural conditions below or above the soil surface, the change in patterns of G × E suggest that germination ecology is affected by light intensity in the face of environmental change, and that this varies across species. If seedling recruitment relies on seeds under the soil surface and these are less resilient to temperature fluctuations, then declines in population size will result. However, if seeds at the soil surface are more affected by extreme temperature fluctuations, seeds at the surface will rarely survive long enough to germinate (Walck *et al.*, 2011).

The timing of germination has critical consequences for later life history stages and reproductive success in natural populations (Donohue *et al.*, 2010; Jiménez‐Alfaro *et al.*, 2016). Mediterranean plants, in particular, have limited scope to shift their distributions, and occupy a highly seasonal environment, placing high importance on predicting germination conditions. Given that ongoing climate change is likely to generate warmer climates with more unpredictable seasonality, Mediterranean ecosystems will need to alter the timing of germination to track rain availability, exposing seeds to suboptimal temperatures outside their historical conditions (Giorgi & Lionello, 2008; Lionello & Scarascia, 2018). It is likely that the exposure to novel environmental conditions will alter seed dormancy dynamics by changing bet‐hedging germination decisions, potentially shifting them towards suboptimal strategies that may affect later life history stages (Ooi *et al.*, 2009; Walck *et al.*, 2011). For example, if rain arrives later it will force populations to germinate closer to winter, where colder temperatures may not be favoured for seedling establishment (Kimball *et al.*, 2010). In these scenarios, the among‐population variance in environmental sensitivity that we detected may help populations to track environmental change. Migration from populations that respond more positively to the new conditions can spread beneficial genetic variation to marginal populations, increasing their resilience (Jump & Penuelas, 2005). However, if dispersal ability is limited, as in many Mediterranean species, then conservation efforts may need to conduct translocations to increase species’ ecological resilience.

In this study, we quantified G × E to test for population variation in germination sensitivity as either a change (increase or decrease) in variation among populations at either temperature extreme or a change in relative performance between temperature extremes, creating a performance trade‐off (Agrawal *et al.*, 2010). For five species, we observed changes in the magnitude of population variance between marginal temperature extremes (i.e. high variance at one temperature extreme but low at the other), suggesting that, for these species, germination is more predictable under certain environmental extremes. Ecological resilience of a given species will be reduced when temperature extremes move in the direction where germination is low. Resilience will also be reduced if the variance among populations is reduced, because there will be no differences among populations and they will all perform similarly poorly when exposed to the novel conditions. However, if conditions move in the direction that reduces germination but there is high variation in performance among populations, then the populations that perform best will be more likely to persist and act as sources for recolonization of sites where performance is lower.

Six species showed evidence of performance trade‐offs between temperature extremes, suggesting that different populations will germinate better depending on whether germination temperatures move towards cold or hot extremes. This suggests that some populations performed better at one extreme but also showed reduced performance at the opposite extreme. If different populations perform best at different environmental extremes, then, regardless of the direction of environmental change (towards warmer or cooler germination temperatures), there will be populations at both extremes that perform relatively well and can help to maintain the resilience of the species.

Population variation in environmental sensitivity (G × E) is likely to be caused by differences among populations in genetic variation or in environmental effects that affect how seeds are produced; for example, maternal effects (Cochrane *et al.*, 2015; Peterson *et al.*, 2018). Genetic differences among populations arise via random genetic changes (drift) or local adaptation (Kawecki & Ebert, 2004). If local adaptation creates variation in environmental sensitivity, then populations adapted to areas that regularly experience higher temperatures during the natural germination period will be pre‐adapted to germinate successfully at higher temperatures. On the other hand, populations exposed to periods of higher temperatures during seed maturation (i.e. late spring or early summer) may have produced seeds with maternal effects that allow germination in conditions similar to those experienced by the maternal plants, increasing germination performance at higher temperatures (Donohue, 2005; Galloway, 2005; Cochrane *et al.*, 2015; Wadgymar *et al.*, 2018). For example, in our results, *C. aeolica* showed differences in environmental sensitivity depending on the year that the same populations were sampled (Fig. 4a), suggesting that maternal effects may have created patterns of G × E.

If the patterns of population variation in environmental sensitivity are created by local adaptation, then selection for alleles underlying favourable G × E may allow evolutionary rescue in response to climate change (Nussey *et al.*, 2005; Bell & Gonzalez, 2009; Chevin *et al.*, 2013). By contrast, if maternal effects are largely responsible for such variation, then ecological resilience may be transient and be limited by the extent to which maternal effects can be transmitted to the next generation (Räsänen & Kruuk, 2007; Auld *et al.*, 2010). To date, few studies have estimated G × E to quantify environmental sensitivity, and then related such patterns to natural environments (e.g. Lopez‐Gallego, 2013). Future experiments should therefore combine laboratory with common‐garden crossing experiments to dissect the role of genetic variation in plasticity vs maternal environmental effects in creating variation in environmental sensitivity (Cochrane *et al.*, 2015).

If population variation in germination sensitivity is driven by current environmental patterns, then understanding the link between patterns of G × E in the laboratory and in the natural environment will help predict species’ responses to environmental change (Frederiksen *et al.*, 2005). Comparing temperature during the natural germination period with patterns of G × E in the laboratory showed an overall positive pattern, suggesting that populations from warmer climates had higher tolerances to warmer temperatures but lower tolerances at cooler temperatures. However, this macroclimatic view needs to be augmented with an understanding of the effect of climate change on microclimates and on biotic interactions (Frederiksen *et al.*, 2005; Barga *et al.*, 2017; Tudela‐Isanta *et al.*, 2018). Future experiments should maximize the number of populations sampled while also logging microclimatic temperature data in the natural environments. Including detailed environmental data at different spatial scales is important to understand how population variation arises and whether different species show similar patterns among sites. Such data are important for making generalizations about ecosystem responses to climate change (Urban *et al.*, 2016; Nadeau *et al.*, 2017).

In other ecosystems, climate change has affected species composition by affecting different species differently and altering ecosystem dynamics (Kimball *et al.*, 2010). The current study reveals species‐specific responses to extreme hot and cold temperatures, but with a common trend of G × E between extreme temperatures for eight of the nine species. To generalize from within‐species variation to among‐species variation, and to better understand the consequences of climate change on Mediterranean ecosystems, we need to consider patterns of G × E present both among populations and among species. The prevalence of G × E suggests that, despite species‐specific responses to climate, G × E within each species promotes ecological resilience and persistence, which should reduce rates of change in community composition, slowing (or preventing) extinction and ecosystem failure. However, seed translocation between populations will likely be necessary to maximize the resilience of Mediterranean communities, especially during extreme years.

## Author contributions

AC conceived the study with input from SC. AC and SC collected seeds and conducted experiments. GMW analysed the data and wrote the paper, with important contributions from AC and JRB. All authors gave final approval for publication.

## Supporting information


**Dataset S1** Full data table for germination performance of all species.

**Fig. S1** Site‐by‐environment interactions for sites sampled in different years.
**Methods S1** Details for the implementation of the Bayesian models.
**Table S1** Sampling locations for each species.
**Table S2** Log likelihood ratio tests.
Please note: Wiley Blackwell are not responsible for the content or functionality of any Supporting Information supplied by the authors. Any queries (other than missing material) should be directed to the *New Phytologist* Central Office.
