# Supplementary material for: Population variation in early development can determine ecological resilience in response to environmental change
Source: New Phytol. 2020 Feb 29;226(5):1312–24. doi: 10.1111/nph.16453 (PMC7317736; doi:10.1111/nph.16453)
Supplement: Supplementary file 2 — Fig. S1 Site‐by‐environment interactions for sites sampled in different years. Methods S1 Details for the implementation of the Bayesian models. Table S1 Sampling locations for each species. Table S2 Log likelihood ratio tests. Please note: Wiley Blackwell are not responsible for the content or functionality of any Supporting Information supplied by the authors. Any queries (other than missing material) should be directed to the New Phytologist Central Office. [file NPH-226-1312-s001.pdf]

Population variation in early development can determine ecological resilience in response to environmental change

Greg M. Walter, Stefania Catara, Jon R. Bridle, Antonia Cristaudo

Supporting Information

Article accepted 20 January 2020

Table S1: Additional information on population locations.

Taxa (Family)	District	Site	Coordinates	Elev. mASL
Centaurea aeolica (Asteraceae)	Lipari (ME)	1.Lipari	38°30'52.66"N 14°57'32.51"E	51
	Santa Marina Salina (ME)	2.Salina	38°34'46.52"N 14°48'42.27"E	159
	Lipari (ME)	3.Panarea	38°38'26.14"N 15°03'49.08"E	338
Erysimum etnense (Brassicaceae)	Ragalna (PA)	1.Mt. Vetore	37°41'34.00"N 14°58'45.03"E	1739
	Nicolosi (CT)	2.Ragala	37°37'26.72"N 15°02'30.29"E	728
Euphorbia characias (Euphorbiaceae)	Linguaglossa (CT)	1.Linguaglossa	37º50'17.32"N 15º06'50.16"E	677
	Francavilla di Sicilia (ME)	2.Francavilla di Sicilia	37º54'40.22"N 15º04'21.41"E	570
Euphorbia dendroides	Castelmola (ME)	1.Castelmola	37º51'40.30"N 15º16'31.26"E	470
	Palermo (PA)	2.Capo Gallo	38º12'58.49"N 13º19'15.50"E	18
	Palermo (PA)	3.Mt. Pellegrino	38º11'40.70"N 13º20'17.08"E	39
Euphorbia rigida	Bronte (CT)	1.Bronte	37º50'56.26"N 14º49'57.99"E	817
	Castiglione di Sicilia (CT)	2.Castiglione di Sicilia	37º51'15.62"N 15º00'31.75"E	956
	Palermo (PA)	3.Isnello	37º52'24.04"N 14º00'44.31"E	1490
Glaucium flavum (Papaveraceae)	Messina (ME)	1.Messina	38°16'20.2"N 15°38'07.2"E	2
	Custonaci (TP)	2.Cofano	38°06'18.6"N 12°41'44.1"E	8
	Erice (TP)	3.Erice	38°02'58.3"N 12°33'08.1"E	8
Jacobaea maritima subsp. *bicolor* (Asteraceae)	Santa Marina Salina (ME)	1.Salina	38°34'13.30"N 14°52'12.80"E	92
	Lipari (ME)	2.Stromboli	38°47'39.28"N 15°13'31.56"E	454
	Lipari (ME)	3.Panarea	38°38'26.14"N 15°03'49.08"E	338
	Lipari (ME)	4.Porticello	38°31'15.93"N 14°57'34.98"E	60
	Lipari (ME)	5.Vulcano	38°22'11.57"N 14°59'39.51"E	20
	Milazzo (ME)	6.Milazzo	38°15'37.07"N 15°14'17.78"E	46
Matthiola fruticulosa (Brassicaceae)	Polizzi Generosa (PA)	1.Quacella	37°50'42.78"N 14°00'56.36"E	1392
	Monreale (PA)	2.Cozzo di Fratantoni	37°59'48.75"N 13°14'47.37"E	1032
Silene fruticose (Caryophyllaceae)	Palermo (PA)	1.Mt. Pellegrino	38°10'55.3"N 13°20'20.8"E	307
	Monreale (PA)	2.Cozzo di Fratantoni	37°59'48.75"N 13°14'47.37"E	1032
	Erice (PA)	3.Erice	38°02'07.05"N 12°35'21.2"E	724
	Palermo (PA)	4.Collesano	37°55'24.96"N 13°56'21.67"E	496
	Palermo (PA)	5.Gratteri	37°58'13.00"N 13°58'35.5"E	666
	Castellammare del Golfo (TR)	6.Mt. Inici	38°01'09.83"N 12°52'13.16"E	301

Table S2: Chi-squared statistics (with degrees of freedom in parentheses) for log likelihood test of interactions for each species.

Species	Light-by-temperature (Fig. 2)	Population-by-temperature interactions (Fig. 4)	
		Dark	Light/Dark
Centaurea aeolica	$\chi 2(5) = 316.02$, $p < 0.001$	$\chi 2(9) = 319.54$, $p < 0.001$	$\chi 2(9) = 210.54$, $p < 0.001$
Erysimum etnense	$\chi 2(6) = 167.27$, $p < 0.001$	$\chi 2(4) = 67.26$, $p < 0.001$	$\chi 2(4) = 64.54$, $p < 0.001$
Euphorbia characias	$\chi 2(4) = 67.15$, $p < 0.001$	$\chi 2(3) = 87.48$, $p < 0.001$	$\chi 2(3) = 51.55$, $p < 0.001$
Euphorbia dendroides	$\chi 2(4) = 26.85$, $p < 0.001$	$\chi 2(2) = 72.84$, $p < 0.001$	$\chi 2(2) = 23$, $p < 0.001$
Euphorbia rigida	$\chi 2(3) = 5.03$, $p = 0.17$	$\chi 2(2) = 21.58$, $p < 0.001$	$\chi 2(2) = 1.8$, $p = 0.406$
Glaucium flavum	$\chi 2(3) = 78.42$, $p < 0.001$	$\chi 2(2) = 14.15$, $p < 0.001$	$\chi 2(2) = 176.1$, $p < 0.001$
Jacobaea maritima	$\chi 2(5) = 67.17$, $p < 0.001$	NA	NA
Matthiola fruticulosa	$\chi 2(7) = 253.62$, $p = < 0.001$	$\chi 2(4) = 14.34$, $p = 0.006$	$\chi 2(4) = 23.9$, $p < 0.001$
Silene fruticosa	$\chi 2(4) = 258.45$, $p = < 0.001$	NA	NA

Note: NA = Not Applicable

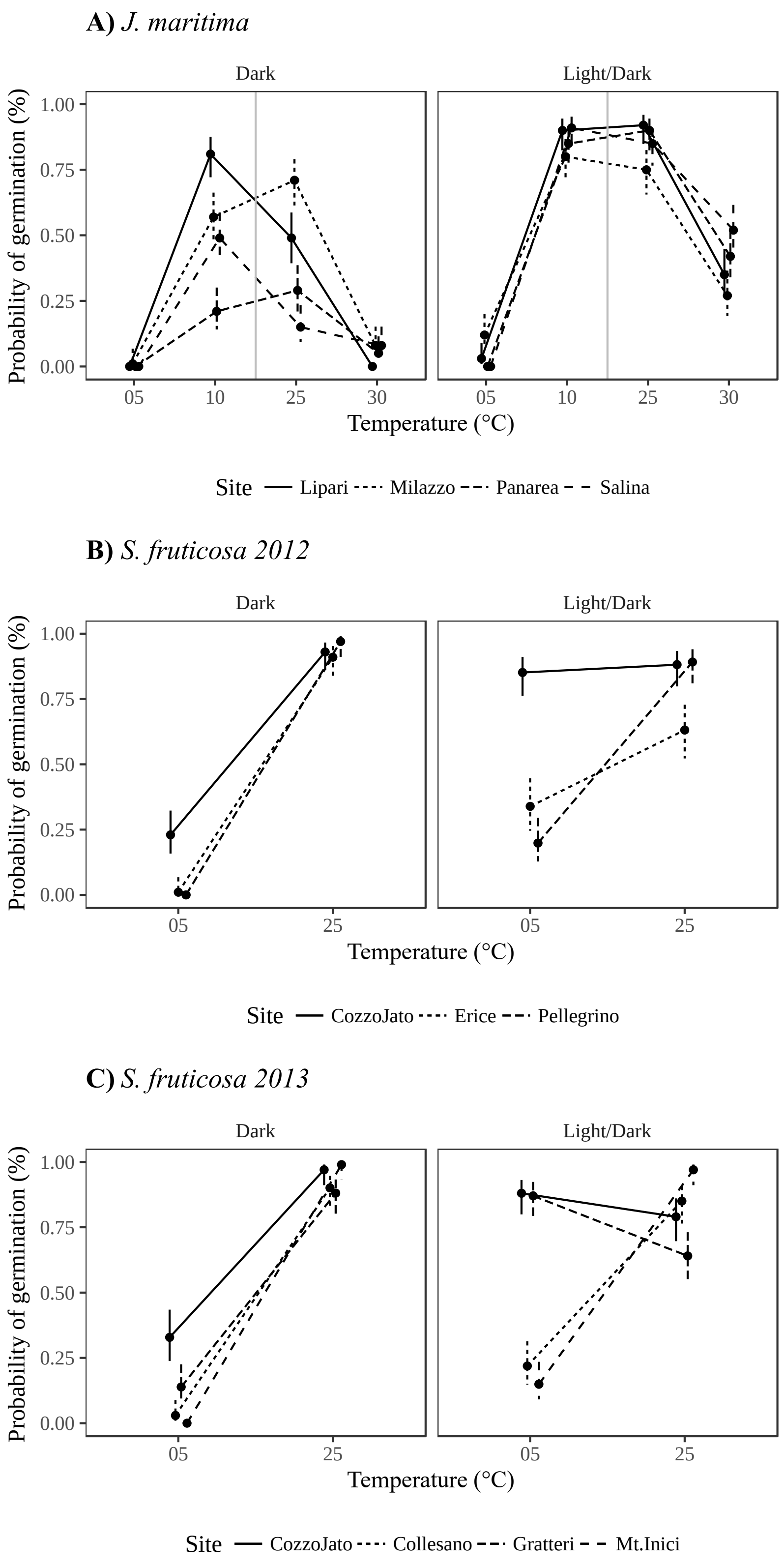

Figure S1: Site-by-environment interactions analysed as fixed effects for sites sampled in the same year (using equation 2) for **A)** *J. maritima*, **B)** *S. fruticosa* sampled in 2012 and **C)** *S. fruticosa* sampled in 2013. Credible intervals represent 95% confidence intervals.

Methods S1

We implemented equation 2 using 13 million Markov Chain Monte Carlo (MCMC) sampling iterations, with a burn-in period of 1.2 million iterations and a thinning interval of 6,500 iterations. We checked model convergence by ensuring effective sample sizes exceeded 85% of the number specified and that autocorrelation values did not exceed 0.05. We used a parameter-expanded prior and checked its sensitivity by changing the scale parameter, ensuring the posterior distribution did not change dramatically. We then extracted 2,000 MCMC iterations from the model output, which provided the posterior distribution for the subsequent analyses. We also extracted the Best Linear Unbiased Predictors (BLUPs) for the performance of each site, in each temperature, which we used to visualize site-by-temperature interactions. Only one parameter showed high autocorrelation between MCMC samples: this was the estimate of germination performance at 5°C for *J. maritima* with a light:dark photoperiod, and consequently we interpreted this parameter with caution.

Dataset S1: Full data table for germination performance of all species (see separate file)
